# Robustness Improvement of Visual Templates Matching Based on Frequency-Tuned Model in RatSLAM

**DOI:** 10.3389/fnbot.2020.568091

**Published:** 2020-09-25

**Authors:** Shumei Yu, Junyi Wu, Haidong Xu, Rongchuan Sun, Lining Sun

**Affiliations:** School of Mechanical and Electrical Engineering, Soochow University, Suzhou, China

**Keywords:** simultaneous localization and mapping, RatSLAM, frequency-tuned model, visual templates, salient map

## Abstract

This paper describes an improved brain-inspired simultaneous localization and mapping (RatSLAM) that extracts visual features from saliency maps using a frequency-tuned (FT) model. In the traditional RatSLAM algorithm, the visual template feature is organized as a one-dimensional vector whose values only depend on pixel intensity; therefore, this feature is susceptible to changes in illumination intensity. In contrast to this approach, which directly generates visual templates from raw RGB images, we propose an FT model that converts RGB images into saliency maps to obtain visual templates. The visual templates extracted from the saliency maps contain more of the feature information contained within the original images. Our experimental results demonstrate that the accuracy of loop closure detection was improved, as measured by the number of loop closures detected by our method compared with the traditional RatSLAM system. We additionally verified that the proposed FT model-based visual templates improve the robustness of familiar visual scene identification by RatSLAM.

## Introduction

In the past 30 years, traditional simultaneous localization and mapping (SLAM) algorithms based on probabilistic mathematical models, such as extended Kalman filter-based SLAM (EKF-SLAM) and Fast-SLAM (Thrun et al., 2005; Lv et al., 2014), have achieved remarkable results. However, the huge number of calculations, high complexity, and large mapping errors of these methods remain to be solved in the field of robot navigation.

Compared with filter-based SLAM (Huang et al., 2013; Srivatsan et al., 2018) and optimization-based SLAM (Hess et al., 2016), the “SLAM” performed in animals demonstrates perfect biological rationality and high adaptability to complex environments; for example, rodents such as mice have powerful navigational capabilities and can solve the entire SLAM problem, even in mazes with crossing paths. Studies have revealed that the biological maps constructed by animals depend on robust processing strategies using place cells, head direction cells, and grid cells, rather than on a precise description of the world (James, 2001). Several researchers have explored the mechanisms underlying rodent navigation using physiological experiments (Hu et al., 2016; Sanchez-Andres et al., 2020), and have subsequently created models of the rodent brain (Fleischer and Edelman, 2009; Krichmar, 2018; Tang et al., 2018) or of memory function (Tan et al., 2013; Tang et al., 2017; Madl et al., 2018; Tang and Michmizos, 2018). Among them, the RatSLAM algorithm based on rodents is widely accepted due to its strong biological rationality and low requirements for computing power (Milford et al., 2004; Milford and Wyeth, 2008; Yuan et al., 2015). Tang et al. (2018) created an episodic memory model inspired by the cornu ammonis 3 (CA3) area of the brain consisting of a recurrent network composed of spiking neurons, which were based on the spike response model. Zou and Cong (2019) proposed using state neurons with high-dimensional information of perception and spatial location, and created a mathematical model expressing the episodic events that the rodent has experienced. However, for complex environments, especially those with varying illumination intensity, RatSLAM, a purely visual brain-inspired SLAM algorithm, has problems such as the low reliability of its visual odometer and its low image matching accuracy.

Several methods have been proposed to address the problems of low matching efficiency and mismatching of visual templates. Milford and Wyeth (2011) proposed a process of visual expectation, which dynamically modifies the recognition thresholds based on recently matched templates, to improve recall performance. Bian et al. (2018) and Shim et al. (2014) proposed adding depth information to the visual template to enhance the robustness of the visual template, which significantly improved the mapping results. Oriented FAST and rotated BRIEF (ORB) is an efficient descriptor with a rotationally invariant character that is suitable for recognizing sequences of familiar visual scenes. Zhou et al. (2017) proposed using the ORB algorithm to extract RGB image features and completing the matching of feature descriptors to improve processing speed and matching accuracy. Srividhya et al. (2019) used speeded-up robust features (SURF), another useful visual processing algorithm, to describe local features. Since the scale-invariant feature transform (SIFT) is highly invariant to scaling and rotation of images, the extracted features are hardly affected by camera tilt (Hu and Liu, 2019). However, setting the threshold of template matching for different scenes is not an easy task, and mismatches may occur from using an improper threshold. To solve this problem, Li et al. (2017) proposed a dynamic threshold-RatSLAM (DT-RatSLAM) method with dynamic threshold adjustment to address mismatching in continuous scenes. Glover et al. (2010) studied the problems that appearance-based mapping and positioning methods face at different times of the day, and fused the RatSLAM and fast appearance based-map (FAB-MAP) methods to create a more robust system in visually varying environments.

If the camera shakes from time to time while collecting data, the reliability of the visual odometer of the system will be greatly reduced. In view of the low reliability of the visual odometer, Adolfsson (2017) tested a robot operating system (ROS) version of Open RatSLAM, which used Viso2 as the visual odometer to generate a robust estimation of motion. Zhang and Hu (2015) introduced information from an optical dual-axis sensor and a micro inertial measurement unit (MIMU) into the visual odometer module of RatSLAM, which improved its accuracy. Shim et al. (2014) used the odometer data provided by the mobile base station to obtain a location estimation, instead of calculating angles, and displacement from image information.

Although the performance of the visual odometer and of image matching can be improved using the methods proposed above, the speed and precision of visual information processing are still unsatisfactory compared with “algorithm” that exists in rodents. The frequency-tuned (FT) model used in this paper simulates the visual information processing of the human optic nerve. We used this model to enhance specific features of the images collected with an Osmo Pocket (an action camera), in order to improve the accuracy of image matching. We expected more robust visual templates to be generated by the FT model, which extracts saliency maps from raw RGB images.

The rest of the paper is organized as follows. Section Traditional RatSLAM details the traditional RatSLAM algorithm. Section Frequency-Tuned Model-Based RatSLAM describes the function of saliency models, then highlights the FT model and visual templates matching based on FT model. Section Experiments and Analysis demonstrates experimental results and analysis to validate the effectiveness of our proposed method. Finally, section Conclusion and Future Work concludes the contribution of this paper, and the shortcomings to be overcome in future work.

## Traditional RatSLAM

**Figure 1:**
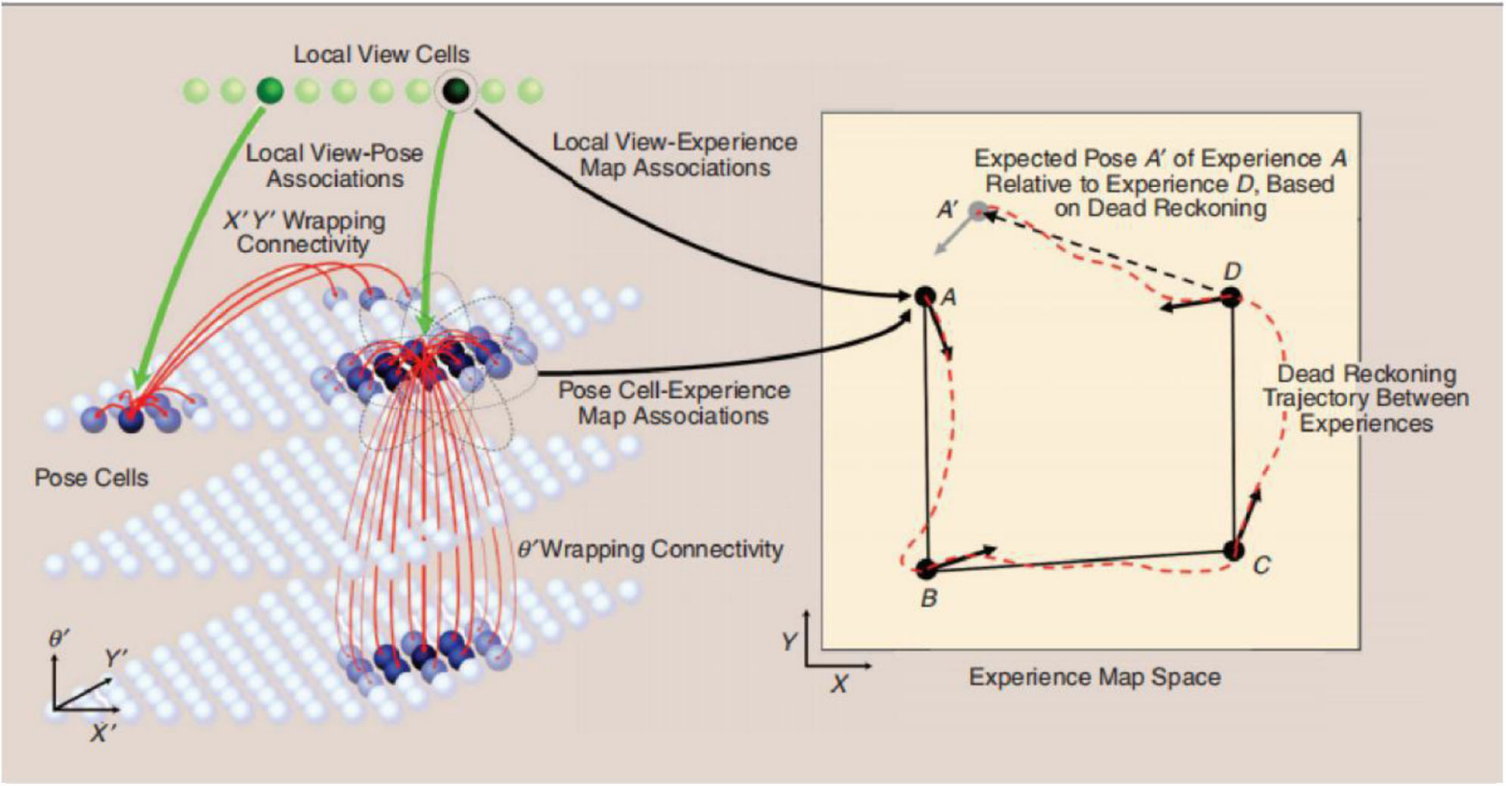
Schematic diagram of the RatSLAM system, courtesy of Jeffery (2007).

The traditional RatSLAM model is primarily composed of local view cells, a pose cell network, a visual odometer, and an experience map. Features in local view cells are generated by summing and normalizing the intensity of all columns of a grayscale image into one-dimensional vectors. The pose cell network aims to represent the position of the robot and its orientation in three-dimensional space. The visual odometer is obtained by comparing the scanning intensity distribution of specific areas in each pair of successive pictures. Finally, local view cells, the pose cell network, and the visual odometer are fused to obtain a two-dimensional cognitive map, as shown in Figure 1 (Wyeth and Milford, 2009).

### Local View Cells

Each local view cell is associated with a cell unit in the continuous attractive network (CAN) model (Milford et al., 2004). In the process of visual template matching, if the current visual template is sufficiently similar to the stored view template, it will inject energy into the relevant units in the CAN model, which is critical for pose cell network correction. Otherwise, if the visual template differs from the stored template, it is transformed into a new template and added to the template library.

### CAN Model

Taking its inspiration from the place cells, head direction cells, and grid cells of the human brain, the CAN model has been adopted as a pose cell model (Jauffret et al., 2012; Knierim, 2015). The function of the CAN model is to ensure the dynamic stability of the pose cells (Shipston-Sharman et al., 2016). Its dynamic process goes through three stages: excitatory update, inhibition, and normalization. During the first stage, each pose cell exerts the excitatory effect on the other pose cells in the pose cell network. In the inhibition stage, each pose cell suppresses the activity of its surrounding cells, which contributes to the convergence of the pose cell network. Finally, the normalization stage maintains the total activity of the pose cells after the visual input and the input of the visual odometer have been obtained.

### Visual Odometer

In the visual odometer, the moving speed and rotational angle information are obtained by comparing the scanning intensity distribution of specific areas in the two images obtained before and after moving. The scanning intensity distribution is a one-dimensional vector, which is obtained by summing the intensities of each column of the grayscale image and normalizing them. The difference between this vector and the one-dimensional vector of the visual template is that they are based on different areas of the images. The activity of pose cells is affected by the visual odometer via path integration (McNaughton et al., 2006).

### Experience Map

The experience map generated in RatSLAM is a topology map representing the paths that the robot has experienced (Milford et al., 2004). Each point in the experience map consists of a pose cell, a local view cell, and the corresponding position in the map. The RatSLAM system compares the current position with those previously stored in the experience map; if the distance is greater than a certain threshold, a new experience will be created. Otherwise, the RatSLAM system detects a loop closure and modifies all experience points to reduce the impact of accumulated errors.

### An Unsolved Problem: The Insufficient Robustness of Visual Templates

**Figure 2:**
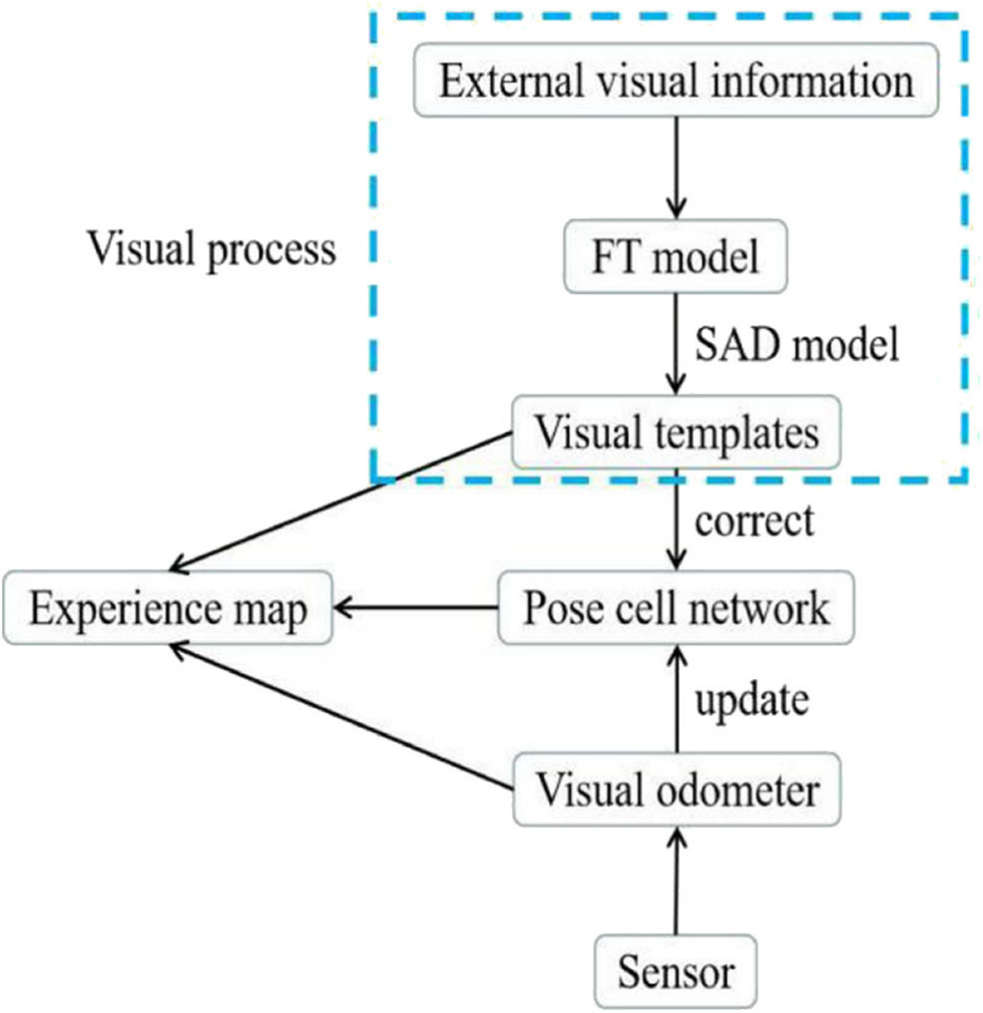
System architecture of FT model-based RatSLAM.

Due to the cumulative error of the visual odometer, RatSLAM needs to correct the experience map by detecting loop closures based on visual template matching; therefore, the accuracy of loop closure detection depends on the visual templates. In traditional RatSLAM, the visual template is obtained by summing and normalizing the intensity of every column of a grayscale image into a vector. The visual template generated by RatSLAM is completely dependent on the pixel intensity and is therefore susceptible to changes in illumination intensity. This means that the error between adjacent templates may become overly large when performing visual template matching, even when their corresponding images are similar. In this paper, to improve the robustness of the visual template, we propose optimizing the visual templates by adding the FT model into the visual processing pipeline, as shown in Figure 2. Before converting the collected image into a visual template, specific features in the image are enhanced by the FT model. As a result, the overall outline of the object in the processed image becomes clearer. Furthermore, the visual templates derived from the FT model are able to include more information related to the image features.

## Frequency-Tuned Model-Based RatSLAM

The main function of the saliency model is to enhance specific features of the image. By considering the saliency description and the corresponding visual template matching, we describe in detail here why the FT model as the appropriate choice. Compared to the visual template generated by RatSLAM, which only contains pixel intensity information, the visual template generated by the FT model also contains color information, which makes the objects in the image more prominent. The expected improvement in the robustness of the visual template obtained from the FT model should increase the accuracy of visual template matching and reduce the number of false matches.

### Definition and Function of Saliency

The ability of the human visual system to quickly search for and locate targets of interest in natural scenes is an important mechanism for visual information processing in daily life. This visual attention mechanism is termed visual saliency and offers a number of significant benefits to visual information processing tasks (Hou et al., 2012). There are two major ways in which visual saliency offers advantages: first, it allows limited computing resources to be allocated to more important information in images and videos; second, the introduction of visual saliency during feature extraction is more consistent with the requirements of human visual cognition.

Existing attention mechanism models can be divided into bottom-up models and top-down models. The method of extracting salient regions used in this paper is a bottom-up visual attention model, known as the saliency model (Marques et al., 2006; Fu et al., 2014). The saliency model is data-driven, such that the influence of human judgement is not considered during image selection. The model mimics the process by which objects with distinctive characteristics are selected when people attend to visual information; this occurs because areas of the image with significant features easily attract attention. Visual saliency is based on specific information in the image, including its static features (such as color, brightness, outline, and texture) and its dynamic features (direction of motion, and speed).

### The FT Model

**Figure 3:**
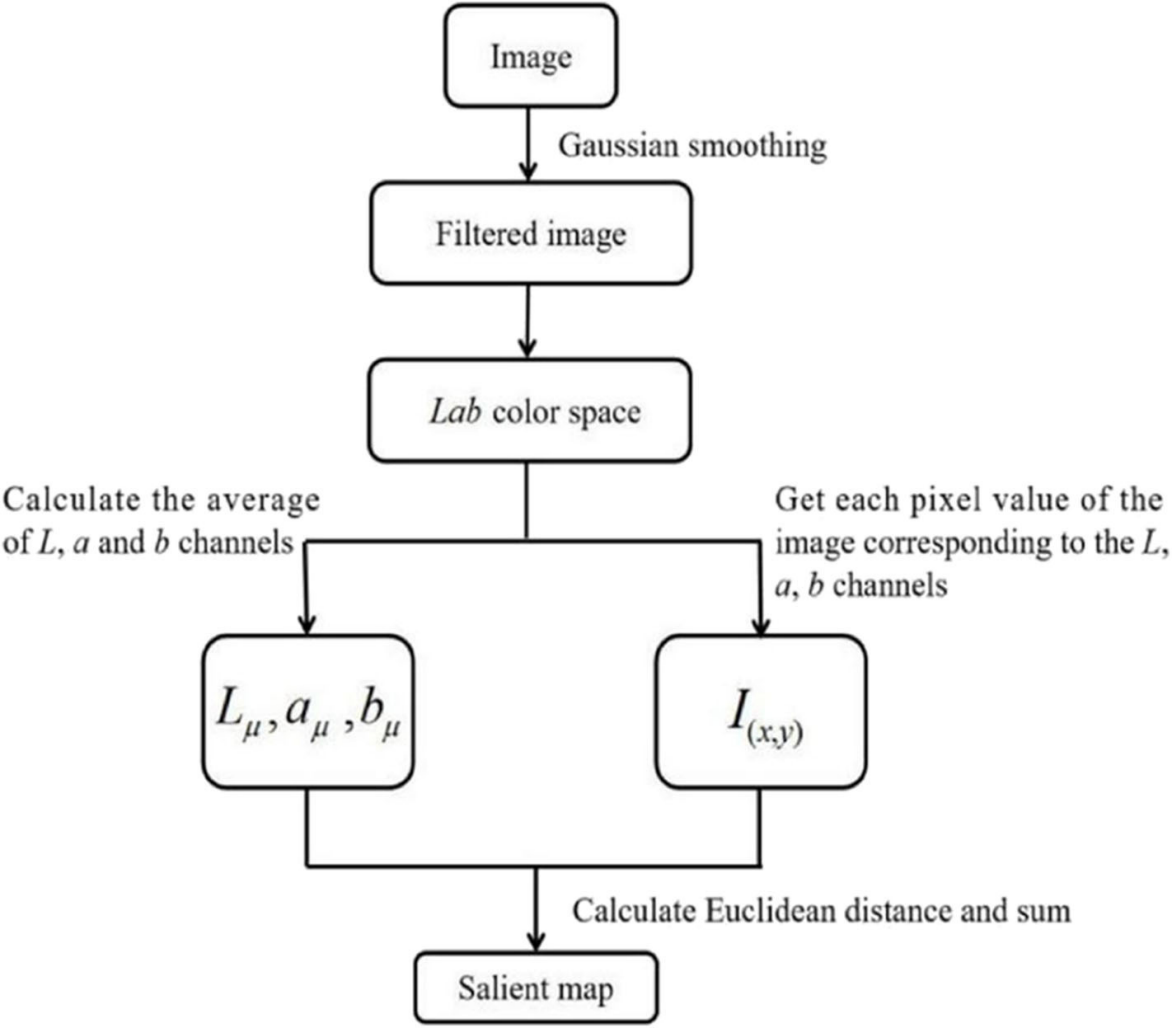
Flow chart of the FT model.

To generate the saliency map, points with larger saliency values are selected as saliency points based on the FT model (Achanta et al., 2009), which processes images in the frequency domain. Generally, the frequency domain of images can be divided into two frequency ranges: high frequency and low frequency. The overall information of the image, such as the outlines of the object, generally belong to the low-frequency range, while the specific details of the image, such as the texture of the object, belong to the high-frequency range. The algorithm used by the FT model can be summarized in the following steps, as shown in Figure 3: (1) Remove the details and noise of the image using a Gaussian smoothing filter; (2) Convert the filtered image from RGB color space to Lab color space to yield the converted image with three channels (*L*, a, and b); (3) Calculate the average values of each channel; (4) For each channel, obtain the Euclidean distance between each pixel value and the average value of the corresponding channel; (5) Finally, for each pixel, sum the values within each of the three channels to build the saliency map.

When Gaussian smoothing is used to filter the image, the selection of the threshold of the low and high frequency ranges is particularly important. In the FT algorithm, w_*lc*_ defines the low-frequency threshold and w_*hc*_ defines the high-frequency threshold. Because a large amount of low-frequency information is present in the salient area of the image, w_*lc*_ is set to a lower value than w_*hc*_ in order to highlight salient objects. In addition, the high-frequency component needs to be retained in order to make the boundary obvious. However, noise and texture often show very high frequencies, so it is necessary to cut off the highest frequencies to avoid interference from noise and texture. In summary, the use of a band-pass filter [w_lc_, w_hc_] is necessary to fulfill these requirements.

The difference of Gaussians (DoG) operator was used to implement the filter function [38]. The formula of the DoG operator is as follows:

(1) $$\begin{array}{r} \begin{array}{c} DoG\left(x,y\right)=\frac{1}{2\pi }\left[\frac{1}{\sigma_{1}^{2}}e^{-\frac{\left(x^{2}+y^{2}\right)}{2\sigma_{1}^{2}}}-\frac{1}{\sigma_{2}^{2}}e^{-\frac{\left(x^{2}+y^{2}\right)}{2\sigma_{2}^{2}}}\right]\\ =G\left(x,y,\sigma_{1}\right)-G\left(x,y,\sigma_{2}\right) \end{array} \end{array}$$

where σ_1_ and σ_2_ are spatial constants that determine the spatial frequency of the image. As shown in Figure 3, the filtered image is then converted into Lab color space and the average of the image in each of the *L*, a, and b channels is calculated to obtain *L*_μ_, a_μ_, and b_μ_. Finally, the generated saliency map *S* is defined as follows:

(2) $$\begin{array}{r} S\left(x,y\right)={\left||I_{\mu }\text{-}I_{whc}\left(x,y\right)|\right|}_{\text{2}} \end{array}$$

where *I*_μ_ is the average value of the Lab space of the original image and *I*_*whc*_(*x, y*) is the pixel value at (*x, y*) of the image. ||.||_2_ is the L2 norm used to calculate the Euclidean distance between *I*_μ_ and *I*_*whc*_(*x, y*) in Lab color space.

**Figure 4:**
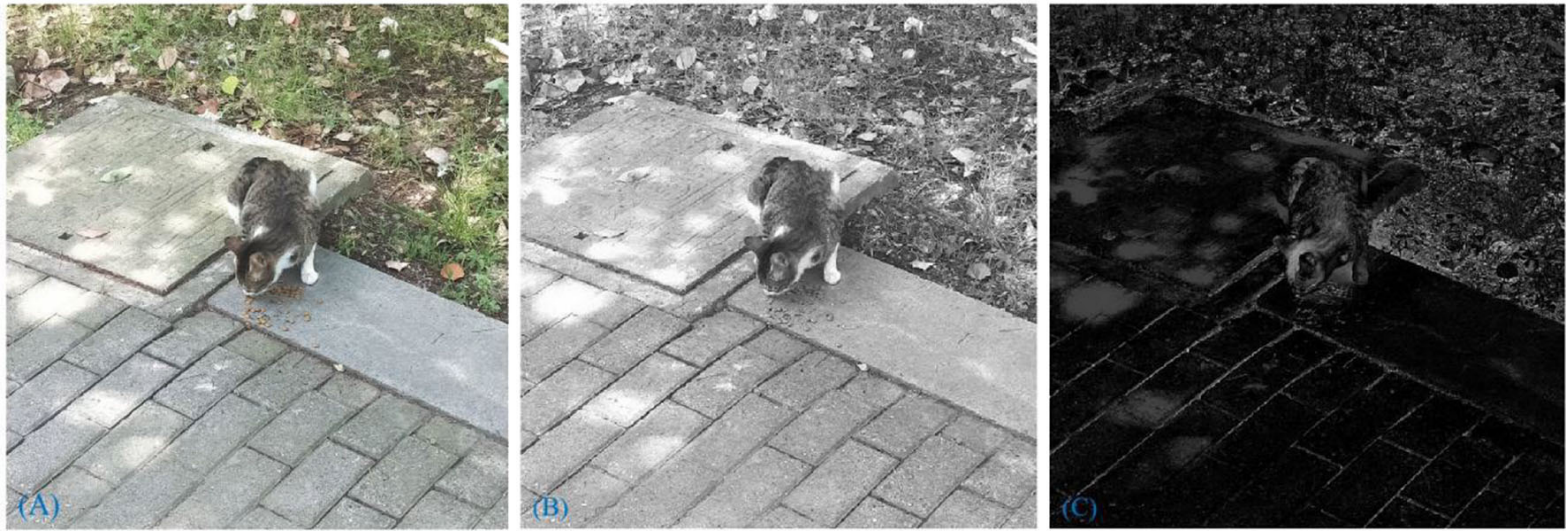
**(A)** Raw picture. **(B)** The grayscale image. **(C)** The picture after saliency model processing.

The FT model has two main advantages: it identifies salient areas with exact boundaries, and outputs saliency maps at full resolution. In Figure 4, for one example image, we compare the corresponding grayscale image with the image generated by the saliency model. This shows that the image processed by the FT model better reflects the boundary of the objects in the image than the image processing used in the traditional RatSLAM algorithm.

### Visual Template Matching

As described above, after processing each image with the FT model, a saliency map is obtained. The saliency map reflects the overall information of the image, such as the outline of the object and the basic areas composing the image. The intensity of each column of the grayscale image is summed and then normalized to obtain the visual template. Finally, the sum of absolute differences (SAD) model (Zhang and Hu, 2015) is used to determine whether the current scene is similar to the stored template. The SAD model is based on the sum of absolute differences and is defined as:

(3) $$\begin{array}{r} f\left(s,I^{j},I^{k}\right)=\frac{1}{w-|s|}\times \left(\overset{w-|s|}{\underset{n=1}{\sum }}|I_{n+\max\left(s,0\right)}^{j}-I_{n+\min\left(s,0\right)}^{j}|\right) \end{array}$$

where *s* is the width of the intensity distribution to be compared, *w* is the width of the visual template, and *I* is a one-dimensional vector obtained from the grayscale image.

The visual templates generated by RatSLAM are not suitable for environments with many similar scenes such as corridors and offices (Zhou et al., 2017). However, visual templates based on the FT model contain color and pixel intensity information, and additionally retain the overall information regarding the objects in the image more comprehensively. Thus, the robustness of the visual templates could potentially be improved based on the FT model.

## Experiments and Analysis

### Experiment Setup

We used four data sets of indoor and outdoor scenes for experimental validation. The first three environmental data sets were collected at a playground, the second floor of a library, and the mini square in front of the Training Building of the Yangcheng Lake Campus, Soochow University, China. An Osmo Pocket camera (DaJiang Innovations, Shenzhen, China) with a field of view (FOV) of 80°, a sampling frequency of 25 Hz, and a resolution of 1,920 × 1,080 was used to collect video data. For the fourth data set, we used the open data set of an office in the Fusionopolis Building, Singapore.

#### Scene 1: A Playground

**Figure 5:**
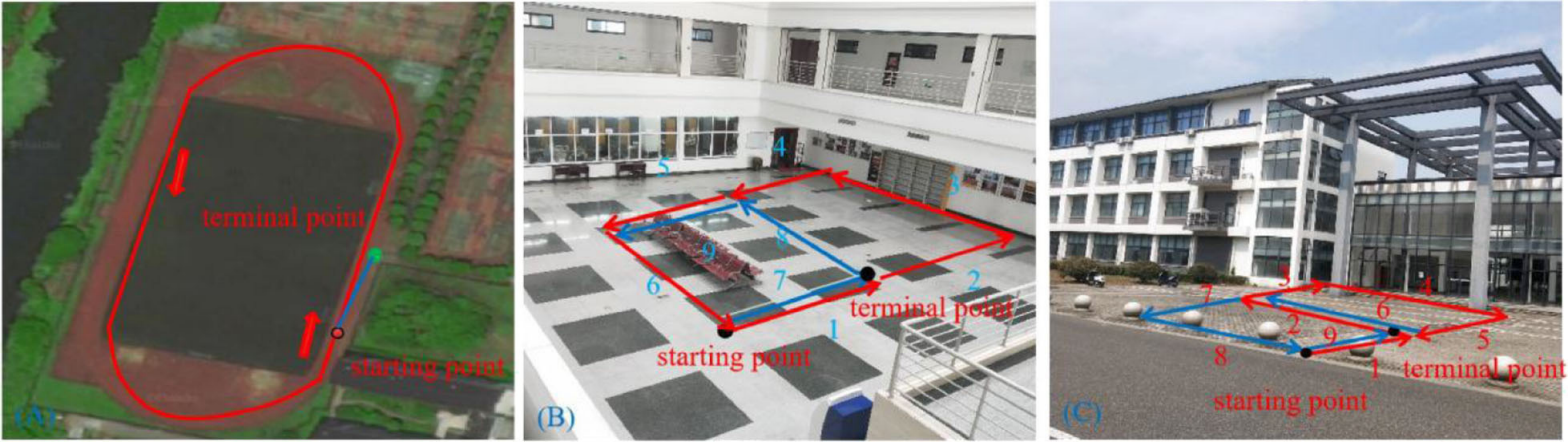
Trajectory diagrams of the experimental environments: **(A)** a playground; **(B)** the second floor of a library; **(C)** the mini square in front of the Training Building, Soochow University, China.

To collect the data, we walked around the 400-m long playground. Importantly, after returning to the starting point, it was necessary to continue walking a short distance (~10 m) along the original route to ensure loop closures in the collected data set. A schematic diagram of the route is shown in Figure 5A.

#### Scene 2: the Second Floor of A Library

This path traversed the second floor of a library in a figure-of-eight-shaped path, as shown in Figure 5B. As with the collection of the playground data set, we continued to walk along the original route a short distance (~10 m) from the starting point to ensure loop closures.

#### Scene 3: the Minisquare in Front of the Training Building

We walked a figure-of-eight-shaped path in the mini square that differed from that of the second scene and additionally, this path was collected outdoors. A schematic diagram of the trajectory is shown in Figure 5C.

#### Scene 4: an Office in the Fusionopolis Building, Singapore

We used an open data set to verify our proposed method. This data set was collected in a typical office scene in Singapore and was provided by Bo. Tian (Shim et al., 2014) in Sichuan University, China.

### Evaluation Criteria

The evaluation criteria used in this paper are precision and recall. The precision is the ratio of the number of true positives (TP) with the sum of TP and the number of false positives (FP). Precision is defined mathematically as:

(4) $$\begin{array}{r} \text{Precision}=\frac{TP}{TP+FP} \end{array}$$

The recall is the ratio of the TP with the sum of TP and the number of false negatives (FN). Recall is defined mathematically as:

(5) $$\begin{array}{r} \text{Recall}=\frac{TP}{TP+FN} \end{array}$$

### Experimental Results

Three data sets were collected with a handheld camera, while the fourth one was an open data set that is publicly available. Our proposed method was run on the four data sets, respectively, to compare it with RatSLAM. We analyzed the experimental results from the perspectives of local view cell activity and of the experience maps generated by the traditional RatSLAM and FT model-based RatSLAM methods. All experimental calculations were performed on a Windows workstation with a Xeon-W2155 3.3 GHz CPU, an Nvidia Geforce RTX2080Ti graphics card, and 128 GB memory. The performance of FT model-based RatSLAM was evaluated by counting the change in the number of visual templates over time and by assessing changes in the experience maps.

#### Local View Cell Activity

**Figure 6:**
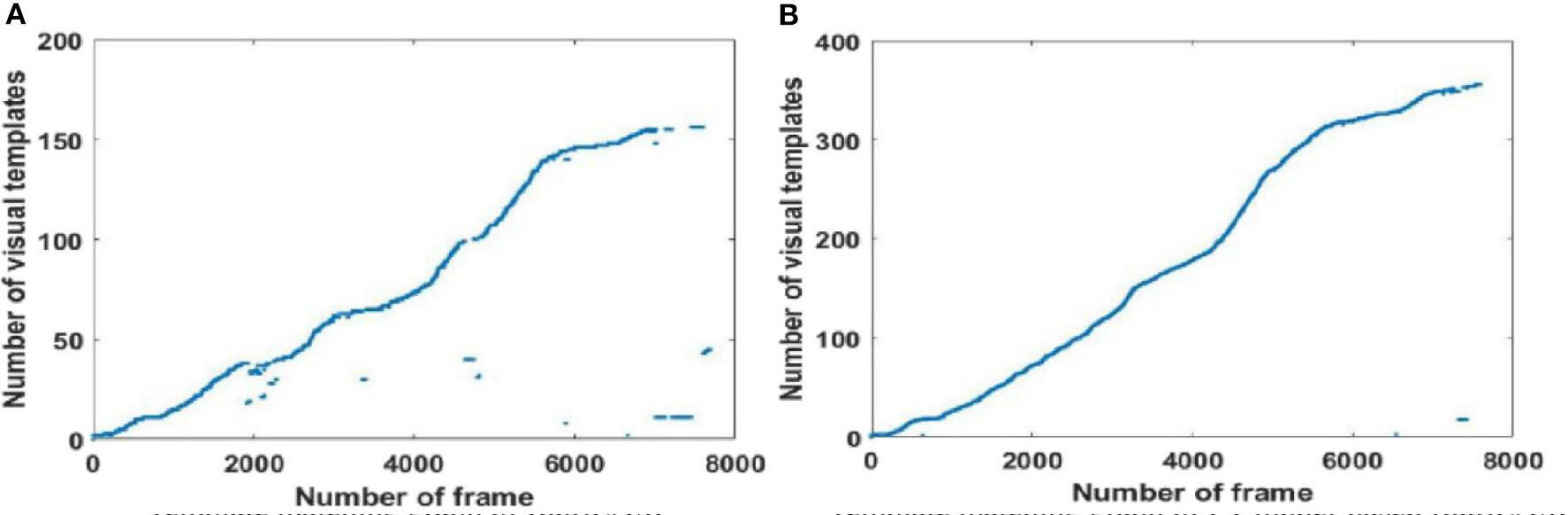
Activity of local view cells during the walk through the playground (Scene 1). **(A)** Template matching graph of RatSLAM. **(B)** Template matching graph of FT model-based RatSLAM.

**Figure 7:**
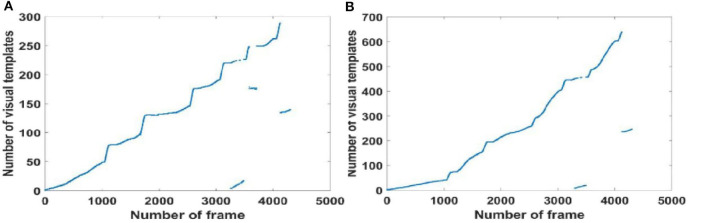
Activity of local view cells during the walk on the second floor of the library (Scene 2). **(A)** Template matching graph of RatSLAM. **(B)** Template matching graph of FT model-based RatSLAM.

**Figure 8:**
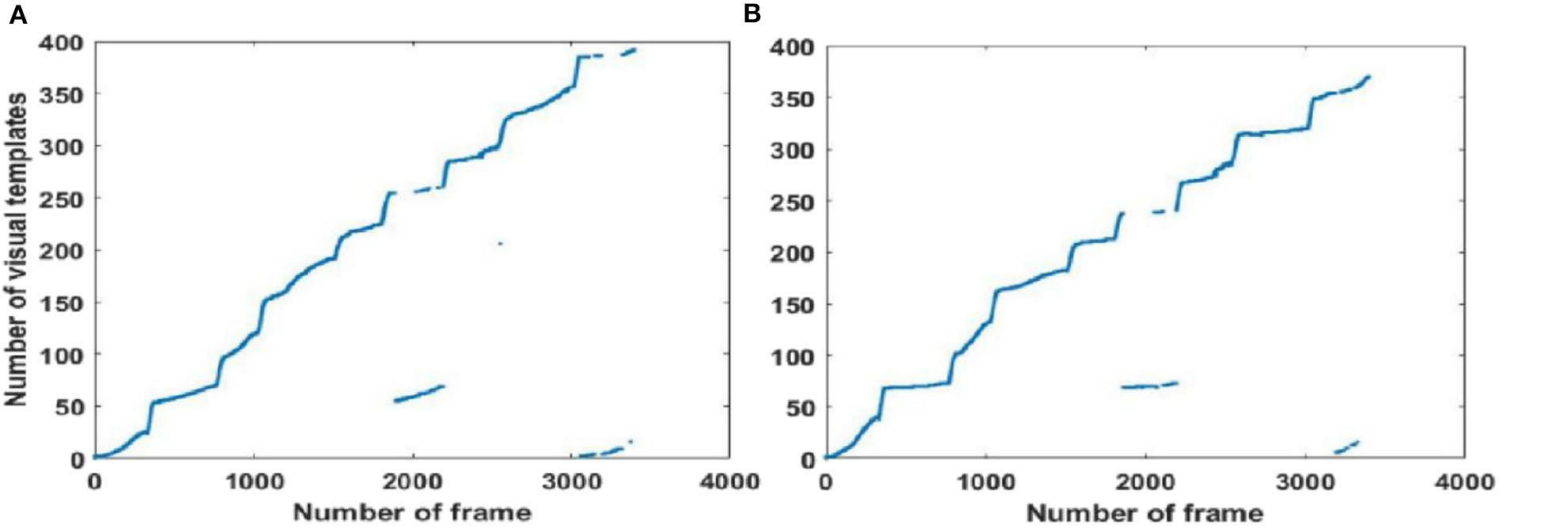
Activity of local view cells during the walk around the mini square in front of the Training Building, Soochow University, China (Scene 3). **(A)** Template matching graph of RatSLAM. **(B)** Template matching graph of FT model-based RatSLAM.

Since visual templates stored in local view cells are used to detect loop closures, which affect the generation of the experience map, we first analyzed local view cell activity. FT model-based RatSLAM compares the current visual scene with the visual templates stored in local view cells. If the degree of similarity exceeds the threshold, the corresponding visual template is considered to be a successful match; otherwise, a new visual template is created. The changes over time in the visual templates obtained from our method are shown in Figures 6–**8**, which correspond to the playground, the second floor of the library, and the mini square in front of the Training Building, respectively. These figures indicate the number of visual templates corresponding to each frame of the video. As shown in Figures 6–8, traditional RatSLAM generated more false matches than FT model-based RatSLAM.

Before returning to the starting point, the number of visual templates should theoretically increase monotonically, with no loop closure because the scenes are always new. As can be seen from Figure 6A generated by traditional RatSLAM, loop closure was detected several times before the return to the starting point; these are false positives that affect the accuracy of the experience map. From Figure 6B based on FT model-based RatSLAM, it can be seen that loop closure is successfully detected upon returning to the starting point, but not that no loop closures are detected before this.

A summarized comparison of the loop closure detection results is given in Table 1. It can be seen from the Scene 1 results that FT model-based RatSLAM not only correctly detects loop closures a greater number of times than traditional RatSLAM, but that it also reduces the number of FP. This is especially important for the accuracy of the experience map, because the error between the generated experience map and the actual path increases as the number of erroneous loop closures increases.

**Table 1:** Comparison of loop closure detection results.

**Dataset**	**Method**	**Expected number of loop closures**	**Number of true positives (TP)**	**Number of false positives(FP)**	**Number of false negatives(FN)**	**Precision**	**Recall**
Scene 1	Traditional RatSLAM	11.0	2.0	12.0	9.0	14.3%	18.2%
	FT model-based RatSLAM	15.0	3.0	1.0	12.0	75.0%	20.0%
Scene 2	Traditional RatSLAM	22.0	12.0	5.0	10.0	70.6%	54.5%
	FT model-based RatSLAM	36.0	22.0	0.0	14.0	100.0%	61.1%
Scene 3	Traditional RatSLAM	64.0	12.0	3.0	52.0	80.0%	18.8%
	FT model-based RatSLAM	70.0	24.0	0.0	46.0	100.0%	34.3%
Scene 4	Traditional RatSLAM	261.0	139.0	17.0	122.0	89.1%	53.3%
	FT model-based RatSLAM	384.0	221.0	2.0	163.0	99.1%	57.6%

In order to further evaluate loop closure detection performance, we chose to walk in a figure-of-eight shape to create a route with loop closures (Scene 2). The route followed is shown in Figure 5, showing that ideally there are two trajectories on which loop closures should be detected, namely path 1 (path 7) and path 5 (path 9) in Figure 5B, and path 1 (path 9) and path 2 (path 6) in Figure 5C.

**Figure 9:**
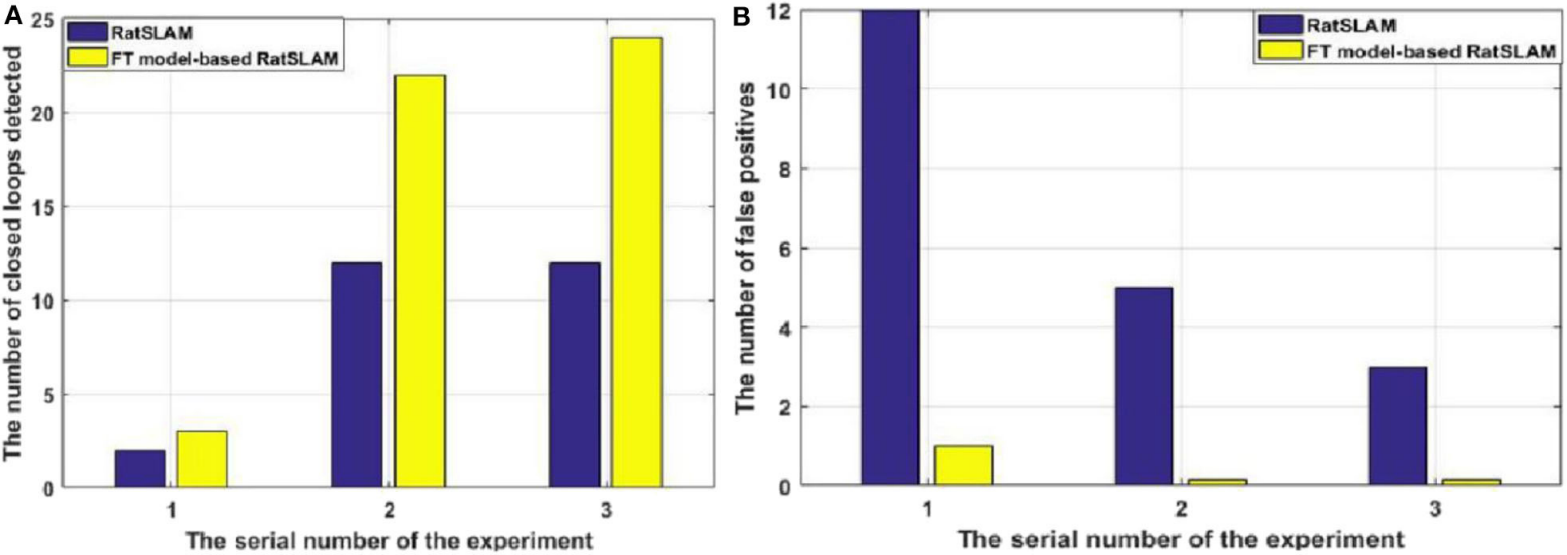
Comparison of the results of the first three experiments. **(A)** Number of loop closures detected. **(B)** Number of false-positive loop closures.

As shown in Figure 7A, traditional RatSLAM detected loop closures in three paths, one of which was unexpected. However, FT model-based RatSLAM detected loop closures in two paths as expected, as shown in Figure 7B. Compared with traditional RatSLAM, the proposed RatSLAM method reduced the number of false-positive loop closures. Similarly, the number of FP of traditional RatSLAM was significantly higher than of FT model-based RatSLAM in Scene 2. As can be seen from Figure 8, traditional RatSLAM only detected loop closure in two of the paths of Scene 3, which matches the actual path. However, the trend in the number of visual templates at around frame 2,000 is relatively flat in Figure 8A, which means that RatSLAM may have detected fewer loop closures than expected. By comparing the loop closures detected in Scene 3, we can see that FT model-based RatSLAM was able to detect more loop closures than the traditional RatSLAM system, which is consistent with our analysis. It can be seen in Figure 9 that traditional RatSLAM produces a greater number of mismatches, while FT model-based RatSLAM reduces the number of FP and improves the matching accuracy.

**Figure 10:**
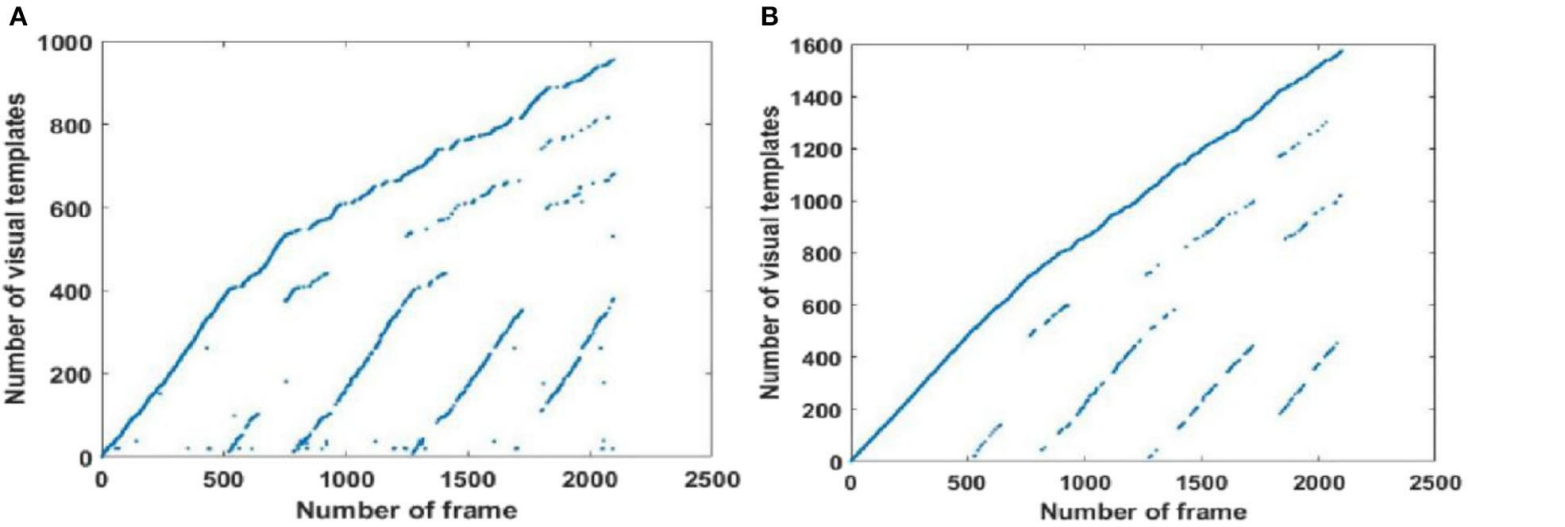
Activity of local view cells during the walk through an office of the Fusionopolis Building, Singapore (Scene 4). **(A)** Template matching graph of RatSLAM. **(B)** Template matching graph of FT model-based RatSLAM.

The open data set from the office in the Fusionopolis Building, Singapore [23] was used to verify our results. The traditional RatSLAM results in Figure 10A show discontinuous points on the graph, indicating a high number of FP. In contrast for the FT model-based RatSLAM results in Figure 10B, there are almost no scattered points, meaning that there were fewer false matches of the visual templates. The results of loop closure detection for the first 1,000 frames are summarized in Table 1. This comparison demonstrates that the FT model does play a role in reducing mismatches.

#### Experience Map Comparison

**Figure 11:**
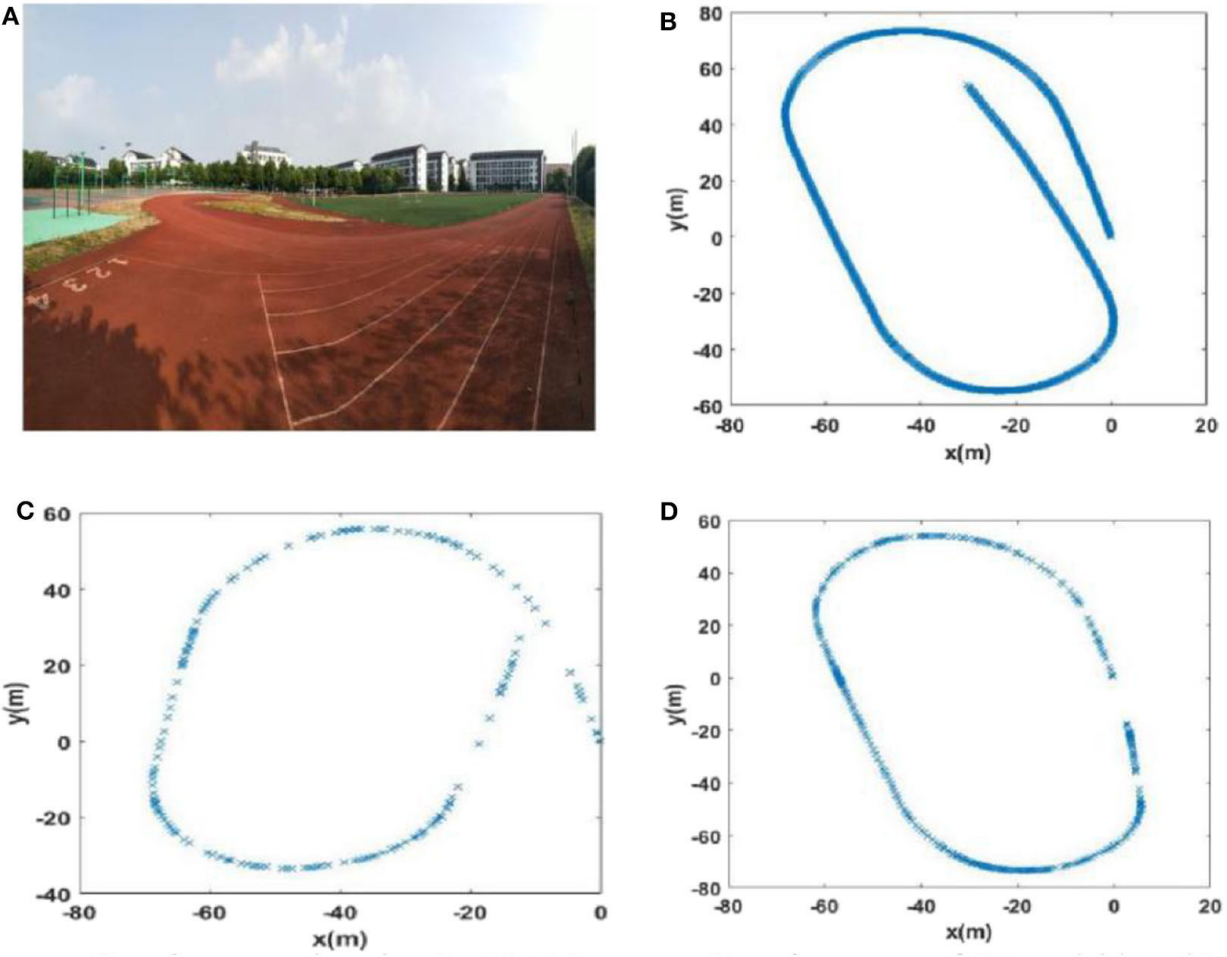
The experience maps generated from the playground data set (Scene 1). **(A)** Experiment environment. **(B)** Visual odomerty. **(C)** Experience map based on RatSLAM. **(D)** Experience map of FT model-based RatSLAM.

**Figure 12:**
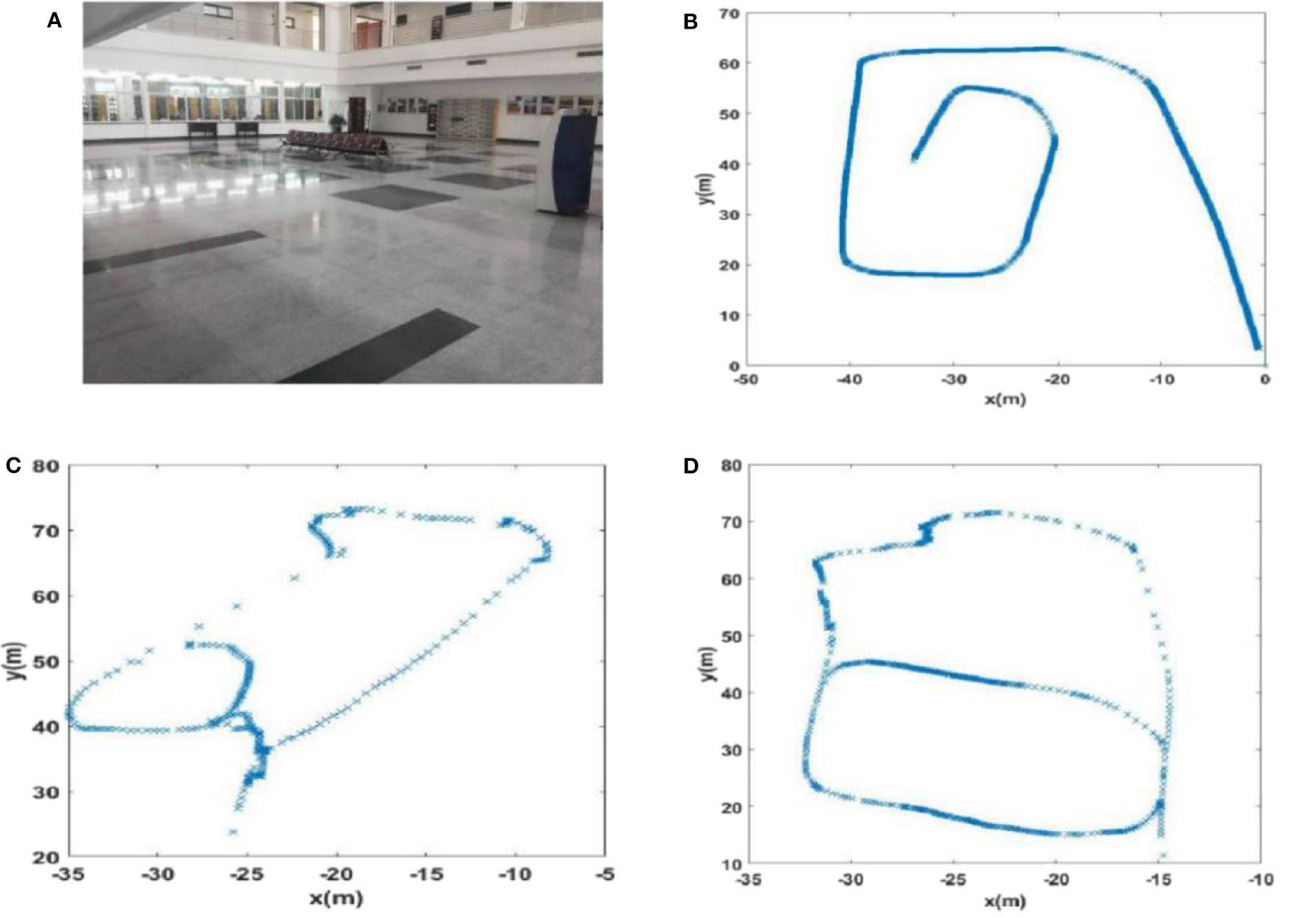
The experience maps generated from the data set of the second floor of the library (Scene 2). **(A)** Experiment environment. **(B)** Visual odomerty. **(C)** Experience map based on RatSLAM. **(D)** Experience map of FT model-based RatSLAM.

**Figure 13:**
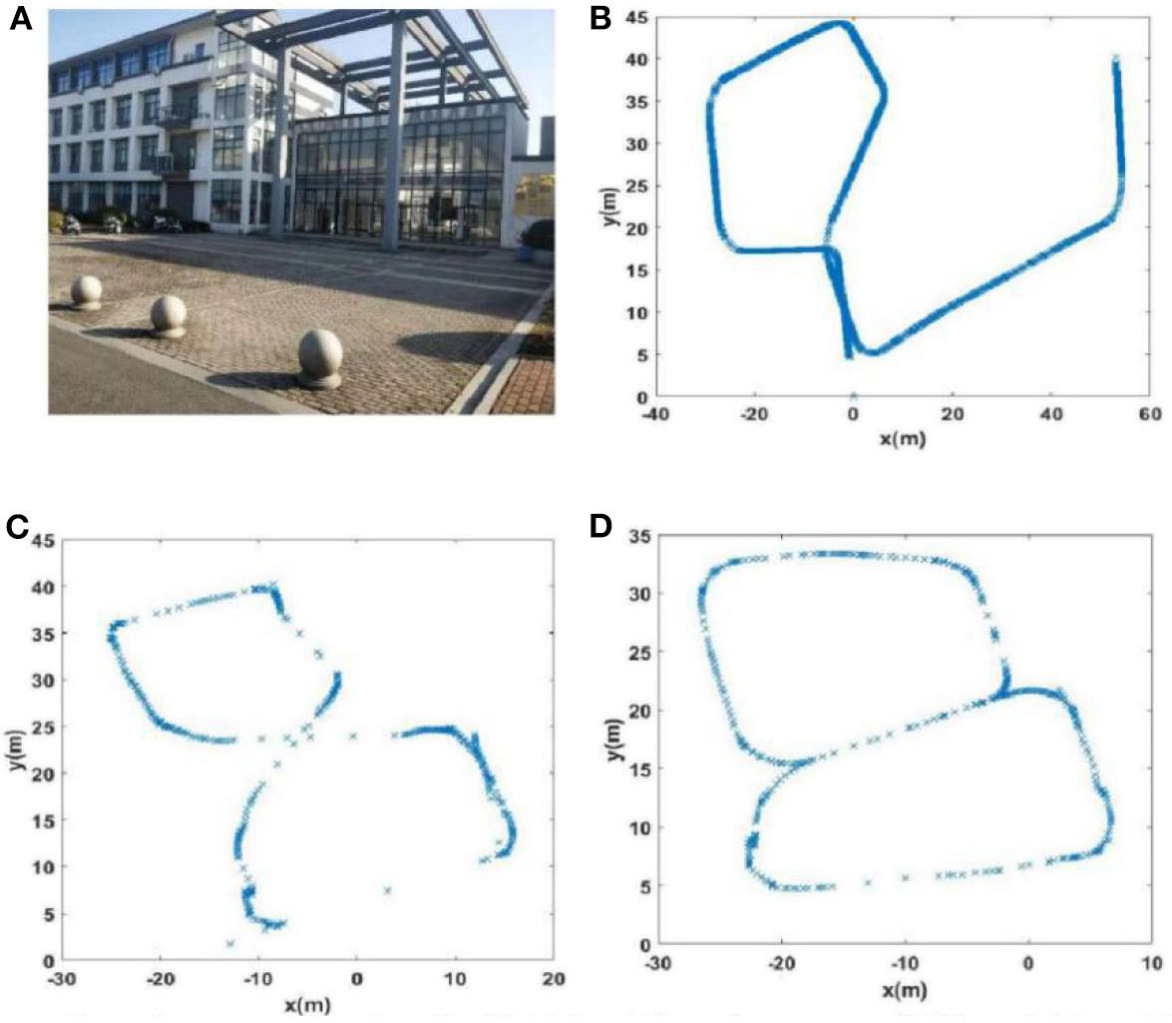
The experience maps generated from the mini square data set (Scene 3). **(A)** Experiment environment. **(B)** Visual odomerty. **(C)** Experience map based on RatSLAM. **(D)** Experience map of FT model-based RatSLAM.

**Figure 14:**
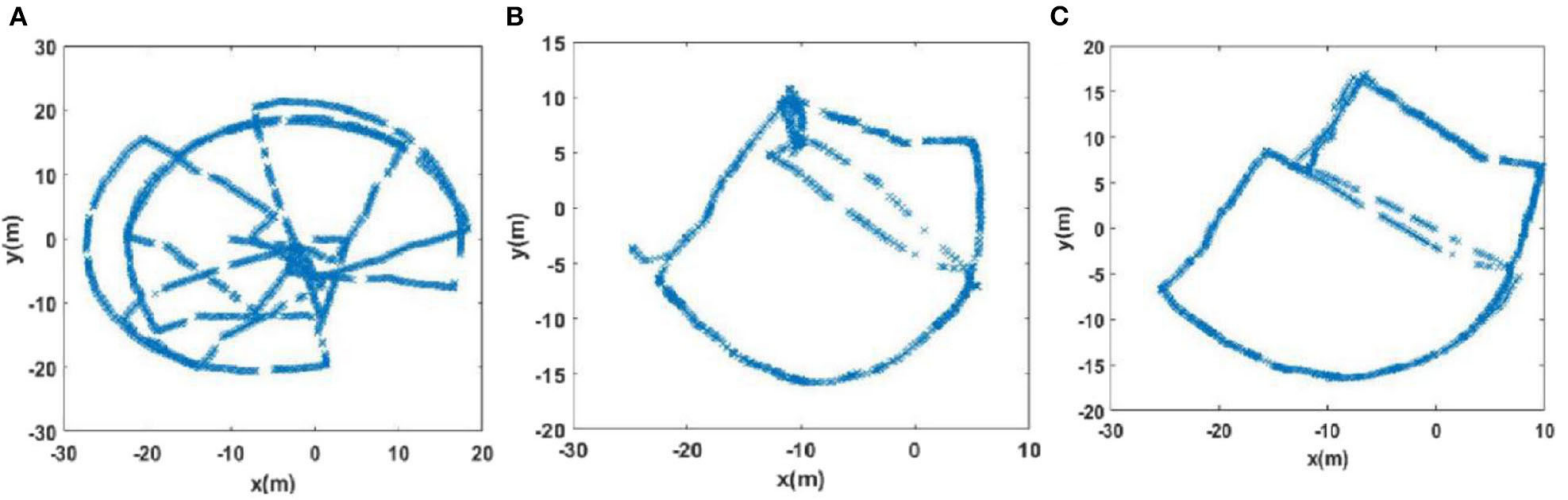
The experience maps generated from the office data set (Scene 4). **(A)** Raw odomerty. **(B)** Experience map based on RatSLAM. **(C)** Experience map of FT model-based RatSLAM.

The experience maps generated by traditional RatSLAM and FT model-based RatSLAM corresponding to Scenes 1, 2, 3, and 4 are shown in Figures 11–**14**, respectively. Comparing the experience maps from the two methods, it can be seen that the FT model-based RatSLAM algorithm based on the saliency model is significantly more adaptable and more robust in complex environments.

Due to incorrect detection of loop closures before returning to the starting point and missing detection of loop closures after returning to the starting point in the playground (Scene 1), the precision and recall of loop closure detection by traditional RatSLAM were only 14.3 and 18.2%, respectively. As such, this method produced an experience map for the playground with large deviation, as shown in Figure 11C. Similarly, it detected several erroneous loop closures, creating a distinct difference between the experience map of the second floor of the library and the actual path, as shown in Figure 12C (Scene 2). As shown in Figure 13C (Scene 3), the experience map generated by traditional RatSLAM detected fewer loop closures than expected, while the experience map in Figure 14B (Scene 4) does now show the expected coinciding of the two paths. In contrast, the experience maps produced by FT model-based RatSLAM are more accurate due to improvements in the precision and recall of loop closure detection, as shown in Figures 11D, 12D, 13D, 14C. These experience maps are more consistent with the actual path traversed during data collection, which is due to the marked effect of the proposed algorithm on loop closure detection. In particular, it is worth noting that the precision of loop closure detection in Scenes 2 and 3 increased to 100%.

As shown in Table 1, compared with traditional RatSLAM, the accuracy of loop closure detection in FT model-based RatSLAM is significantly improved and the recall is also slightly improved. While there is still a certain number of missed detections of loop closures from overlapping paths, this has less of a negative impact on the experience map compared with wrong matches. It is worth mentioning that FT model-based RatSLAM reduces the number of mismatches while ensuring that recall does not decrease; this improvement is crucial to the accuracy of the generated experience map.

## Conclusion and Future Work

In this paper, the saliency model was applied to the RatSLAM system to extract visual templates, in order to improve the robustness of visual templates and the performance of the loop closure detection algorithm. In order to test the effectiveness of the saliency model, its performance was verified by comparing the effects on the experience maps generated in a number of different environments. The experimental results showed that visual templates based on the FT model significantly enhanced the robustness of the brain-inspired SLAM system. These greatly reduced the number of FP, improved the precision and recall of loop closure detection, and resulted in a more accurate experience map.

The experiments showed that the robustness of the visual templates generated by the FT model were indeed improved, as shown by the reduced number of mismatches. However, the experimental analysis indicated several issues that would be worthy of further investigation and improvement. First, there were a number of missed matches because of camera tilt. The extracted features are not rotatable in the same way as ORB features, so matches between similar views may be missed; this has a negative effect on the accuracy of the experience map. To reduce the impact of camera tilts, the camera can be installed on a more stable platform. In addition, the collected images can be made more in line with those sampled by rodents by placing the camera at a lower height on the robot platform, which would make the method more convincing biologically. Second, during the experiment, it was found that environments containing trees are more prone to mismatch, which may relate to the features extracted by the FT model. This indicates that other saliency models may be more suitable for use in environments with trees. The question of which saliency model is most suitable for a given environment is one that is worthy of further discussion. Finally, we only used a bottom-up attention model to extract saliency maps from images in this paper; however, rodents use both bottom-up and top-down attention models in combination when they navigate. Therefore, in future, it will be necessary to explore how navigation can be performed using both of these attention models together.

## Data Availability Statement

All datasets generated for this study are included in the article/supplementary material.

## Author Contributions

SY proposed the idea of saliency model-based visual template matching. JW conducted the experiments, analyzed the experimental results, and wrote the manuscript. RS designed the overall research plan and helped with the manuscript writing. HX helped with data collection and programming. LS provided the experimental location and helped to review the manuscript. All authors contributed to the article and approved the submitted version.

## Conflict of Interest

The authors declare that the research was conducted in the absence of any commercial or financial relationships that could be construed as a potential conflict of interest.

## References

[B1] Achanta R. Hemami S. Estrada F. Susstrunk S. (2009). Frequency-tuned salient region detection, in Proceedings of the IEEE Conference on Computer Vision and Pattern Recognition (Florida, FL), 1597–1604. 10.1109/CVPR.2009.5206596

[B2] Adolfsson S. (2017). RatSLAM with Viso2: implementation of alternative monocular odometer, in Vetenskapliga Arkivet (DiVA) (Digitala), 40.

[B3] Bian C. Y. Ling Y. Z. Cheng M. Y. (2018). Research on RatSLAM bionics algorithm with fusion depth information. J. Shaanxi Univ. Technol. 34, 44–50.

[B4] Fleischer J. Edelman G. (2009). Brain-based devices. IEEE Robot. Autom. Mag. 16, 33–41. 10.1109/MRA.2009.933621

[B5] Fu L. H. Guo L. Wu W. D. (2014). Salient region detection based on frequency-tuning and region contrast, in Proceedings of the 3rd International Conference on Computer Science and Service System, 732–735. 10.2991/csss-14.2014.171

[B6] Glover A.J. Maddern W.P. Milford M. Wyeth G.F. (2010). FAB-MAP+RatSLAM: appearance-based SLAM for multiple times of day, in Proceedings of the IEEE International Conference on Robotics and Automation (ICRA) (Anchorage, AK), 3507–3512. 10.1109/ROBOT.2010.5509547

[B7] Hess W. Kohler D. Rapp H. Andor D. (2016). Real-time loop closure in 2D LIDAR SLAM, in IEEE International Conference on Robotics and Automation (Stockholm), 1271–1278. 10.1109/ICRA.2016.7487258

[B8] Hou X. D. Harel J. Koch C. (2012). Image signature: highlighting sparse salient regions. IEEE Tranc. Pattern Anal. 34, 194–201. 10.1109/TPAMI.2011.146 21788665

[B9] Hu J. Tang H. J. Tan K. C. (2016). How the brain formulates memory: a spatio-temporal model*. IEEE Comput. Intell. M* 11, 56–68. 10.1109/MCI.2016.2532268

[B10] Hu W. Liu X. Y. (2019). A monocular SLAM image matching method based on improved SIFT algorithm. Electron. Opt. Control 26, 7–13. 10.3969/j.issn.1671-637X.2019.05.002

[B11] Huang G. P. Mourikis A. I. Roumeliotis S. I. (2013). A quadratic-complexity observability-constrained unscented Kalman filter for SLAM. IEEE Tranc. Robot. 29, 1226–1243. 10.1109/TRO.2013.2267991

[B12] James O. (2001). How brains make up their mind. Complexity 6:5 10.1002/cplx.1048

[B13] Jauffret A. Cuperlier N. Gaussier P. (2012). Multimodal integration of visual place cells and grid cells for navigation tasks of a real robot, in Proceedings of the International Conference on Simulation of Adaptive Behavior (Odense), 136–145. 10.1007/978-3-642-33093-3_14

[B14] Jeffery K.J. (2007). Self-localization and the entorhinal-hippocampal system. Curr. Opin. Neurobiol. 17, 684–691. 10.1016/j.conb.2007.11.008 18249109

[B15] Knierim J. J. (2015). From the GPS to HM: place cells, grid cells, and memory. Hippocampus 25, 719–725. 10.1002/hipo.22453 25788454

[B16] Krichmar J. L. (2018). A thriving community and a promising pathway toward intelligent cognitive robots. Front. Neurorobotics 12:42. 10.3389/fnbot.2018.00042 30061820 PMC6054919

[B17] Li W. L. Wu D. W. Hu L. (2017). Independent location and mapping method with bionic synchronization based on dynamic threshold. J. Xian Jiaotong Univ. 51, 100–106. 10.7652/xjtuxb201710017

[B18] Lv J. Kobayashi Y. Ravankar A. A. (2014). Straight line segments extraction and EKF-SLAM in indoor environment. J. Automation. Control Eng. 2, 270–276. 10.12720/joace.2.3.270-276

[B19] Madl T. Frankin S. Chen K. (2018). A computational cognitive framework of spatial memory in brains and robots. Cogn. Syst. Res. 47, 47–172. 10.1016/j.cogsys.2017.08.002

[B20] Marques O. Mayron L. M. Borba G. B. (2006). Using visual attention to extract regions of interest in the context of image retrieval, in Proceedings of the 44th Annual Southeast Regional Conference (Melbourne, FL), 638–643. 10.1145/1185448.118558

[B21] McNaughton B. L. Battaglia F. P. Jensen O. (2006). Path integration and the neural basis of the ‘cognitive map'. Nat. Rev. Neurosci. 7, 663–678. 10.1038/nrn1932 16858394

[B22] Milford M. Wyeth G. (2008). Mapping a suburb with a single camera using a biologically inspired SLAM system. IEEE Trans. Robot. 24, 1038–1053. 10.1109/TRO.2008.2004520

[B23] Milford M. Wyeth G. (2011). Improving recall in appearance-based visual SLAM using visual expectation, in Australasian Conference on Robotics and Automation (Brisbane, QLD), 173–1181.

[B24] Milford M. Wyeth G. Prasser D. P. (2004). RatSLAM: a hippocampal model for simultaneous localisation and mapping, in IEEE International Conference on Robotics and Automation (ICRA) (New Orleans, LA), 403–408. 10.1109/ROBOT.2004.1307183

[B25] Sanchez-Andres J. V. Pomares R. Malaisse W. J. (2020). Adaptive short-term associative conditioning in the pancreatic β-cell. Physiol. Rep. 8:e14403. 10.14814/phy2.14403 32232927 PMC7105902

[B26] Shim V. A. Tian B. Yuan M. Tang H. Li H. (2014). Direction-driven navigation using cognitive map for mobile robot, in Proceedings of the IEEE/RSJ International Conference on Intelligent Robots and Systems (IROS) (Chicago, IL), 2639–2646. 10.1109/IROS.2014.6942923

[B27] Shipston-Sharman O.liver. Solanka L. Nolan M. F. (2016). Continuous attractor network models of grid cell firing based on excitatory-inhibitory interactions. J. Physiol. 594, 6547–6557. 10.1113/JP270630 27870120 PMC5108899

[B28] Srivatsan R. A. Xu M. Zevallos N. (2018). Probabilistic pose estimation using a bingham distribution-based linear filter. Int J. Robot Res. 37, 1610–1631. 10.1177/0278364918778353

[B29] Srividhya S. Prakash S. Elangovan K. (2019). 3D reconstruction of an indoor environment using SLAM with modified SURF and A-KAZE feature extraction algorithm, in Proceedings of the International Conference on Intelligent Computing, Information and Control Systems (Xi'an), 133–143. 10.1007/978-3-030-30465-2_16

[B30] Tan C. H. Tang H. Tan K. C. Yuan M. (2013). A hippocampus CA3 spiking neural network model for storage and retrieval of sequential memory, in IEEE Conference on Cybernetics and Intelligent Systems (CIS) (Manila), 134–139. 10.1109/ICCIS.2013.6751592

[B31] Tang G. Michmizos K. P. (2018). Gridbot: an autonomous robot controlled by a spiking neural network mimicking the brain's navigational system, in International Conference on Neuromorphic Systems (ICONS) (NexComm), 4–11. 10.1145/3229884.3229888

[B32] Tang H. Huang W. Narayanamoorthy A. Yan R. (2017). Cognitive memory and mapping in a brain-like system for robotic navigation. Neural Netw. 87, 27–37. 10.1016/j.neunet.2016.08.015 28064015

[B33] Tang H. Yan R. Tan K. C. (2018). Cognitive navigation by neuro-inspired localization, mapping, and episodic memory. IEEE T. Cogn. Dev. Syst. 10, 751–761. 10.1109/TCDS.2017.2776965

[B34] Thrun S. Burgard W. Fox D. (2005). Probabilistic Robotics. MIT Press.

[B35] Wyeth G. Milford M. (2009). Spatial cognition for robots. IEEE Robot. Autom. Mag. 16, 24–32. 10.1109/MRA.2009.933620

[B36] Yuan M. Tian B. Shim V. A. Tang H. Li H. (2015). An entorhinal-hippocampal model for simultaneous cognitive map building, in Proceedings of the 29th Conference of the Association for the Advance of Artificial Intelligence (AAAI) (Austin, TX), 586–592.

[B37] Zhang X. Hu X. P. (2015). An improved bionic navigation algorithm based on RatSLAM. Navig. Control. 14, 73–80. 10.3969/j.issn.1674-5558.2015.05.013

[B38] Zhou S. C. Yan R. Li J. X. (2017). A brain-inspired SLAM system based on ORB features. Int. J. Autom. Comput. 14,564–575. 10.1007/s11633-017-1090-y

[B39] Zou Q. Cong M. (2019). Robotic path planning based on episodic-cognitive map. Int. J. Control Autom. Sys. 17, 1304–1313. 10.1007/s12555-018-0141-7

